# Non-coding RNA alterations in extracellular vesicles from bronchoalveolar lavage fluid contribute to mechanical ventilation-induced pulmonary fibrosis

**DOI:** 10.3389/fimmu.2023.1141761

**Published:** 2023-03-13

**Authors:** Ri Tang, Yue Hu, Shuya Mei, Yang Zhou, Jinhua Feng, Tao Jin, Bo Dai, Shunpeng Xing, Yuan Gao, Qiaoyi Xu, Zhengyu He

**Affiliations:** ^1^ Department of Critical Care Medicine, Ren Ji Hospital, Shanghai Jiao Tong University School of Medicine, Shanghai, China; ^2^ Shanghai Key Laboratory of Modern Optical System, School of Optical-Electrical and Computer Engineering, University of Shanghai for Science and Technology, Shanghai, China

**Keywords:** non-coding RNA, extracellular vesicles, mechanical ventilation, pulmonary fibrosis, bioinformatics analysis

## Abstract

**Objective:**

For respiratory failure patients, mechanical ventilation (MV) is a life-saving therapy to maintain respiratory function. However, MV could also cause damage to pulmonary structures, result in ventilator-induced lung injury (VILI) and eventually progress to mechanical ventilation-induced pulmonary fibrosis (MVPF). Mechanically ventilated patients with MVPF are closely related to increased mortality and poor quality of life in long-term survival. Thus, a thorough understanding of the involved mechanism is necessary.

**Methods:**

We used next-generation sequencing to identify differentially expressed non-coding RNAs (ncRNAs) in BALF EVs which were isolated from Sham and MV mice. Bioinformatics analysis was conducted to identify the engaged ncRNAs and related signaling pathways in the process of MVPF.

**Results:**

We found 1801 messenger RNAs (mRNA), 53 micro RNAs (miRNA), 273 circular RNAs (circRNA) and 552 long non-coding RNAs (lncRNA) in mice BALF EVs of two groups, which showed significant differential expression. TargetScan predicted that 53 differentially expressed miRNAs targeted 3105 mRNAs. MiRanda revealed that 273 differentially expressed circRNAs were associated with 241 mRNAs while 552 differentially expressed lncRNAs were predicated to target 20528 mRNAs. GO, KEGG pathway analysis and KOG classification showed that these differentially expressed ncRNA-targeted mRNAs were enriched in fibrosis related signaling pathways and biological processes. By taking the intersection of miRNAs target genes, circRNAs target genes and lncRNAs target genes, we found 24 common key genes and 6 downregulated genes were confirmed by qRT-PCR.

**Conclusions:**

Changes in BALF-EV ncRNAs may contribute to MVPF. Identification of key target genes involved in the pathogenesis of MVPF could lead to interventions that slow or reverse fibrosis progression.

## Introduction

Among prolonged acute respiratory distress syndrome (ARDS) patients, pulmonary fibrosis has become a leading cause of death. For years after ARDS, most survivors experience poor health-related quality of life due to fibroproliferative responses which characterized by collagen deposition and fibroblast accumulation in pulmonary tissue (1). To ARDS patients, mechanical ventilation (MV) is the most crucial supportive therapy. However, MV is a double-edged sword that may further aggravate the damage of ARDS lungs. In spite of the widespread promotion and use of low tidal volume ventilation, cyclic mechanical stretch may still contribute to ventilation-induced pulmonary injury (VILI) (2–4) and mechanical ventilation-induced pulmonary fibrosis (MVPF) (5–7). A complete understanding of the mechanisms involved in MVPF is not yet available.

Extracellular vesicle (EV) is one of the means of intercellular communication between adjacent and distant cells by its containing cargoes such as nucleic acids and proteins (8). Non-coding RNAs (ncRNA) in EVs have been shown to be taken up by neighboring or distant cells and subsequently modulate the recipient cells (9). However, few studies have examined the role of EV and its contained ncRNAs in MVPF. In this study, we researched changes in ncRNA expression in BALF EVs of MV-treated mice by high‐throughput whole transcriptome sequencing. Through analysis of ncRNA target mRNAs and functional enrichment associated with fibrosis through bioinformatic methods, we explored the possible role of BALF-EV ncRNAs in MVPF. By taking the intersection of messenger RNA (miRNA) target genes, circular RNA (circRNA) target genes and long non-coding RNA (lncRNA) target genes, 24 common key genes were found and the expression of 6 genes were proved to be downregulated by qRT-PCR. Our findings shed some light on the role of BALF-EV ncRNAs in the process of MVPF and may lead to potential interventions for MVPF in the future.

## Materials and methods

### Ethics statement and animals

C57BL/6 Male mice (6-8week; 18–27g) were acquired from Shanghai SLAC Laboratory Animal (Shanghai, China). The animals were kept in a controlled environment with a temperature of 22-24°C, a 12h light/dark cycle, and free access to food and water. The Animal Care and Use Committee of Ren Ji Hospital, Shanghai Jiao Tong University School of Medicine approved all experiments.

### MV model and animal procedures

C57BL/6 Male mice (6-8week; 18–27g) were anesthetized with intraperitoneal injection of 200 mg/kg Ketamine, and 10 mg/kg Xylazine, and divided into Sham group (n=6) and MV group(n=6) randomly. Mice in the MV group were mechanically ventilated for 2h using FiO2 0.21, VT 20ml/kg and RR 70 breaths/min (10), while the Sham group kept spontaneous breath after intubation. Following intubation, the animals were returned to the animal facility with free access to water and food and monitored for seven days.

### Pulmonary histopathology

4% paraformaldehyde was used to fix lung tissue overnight, which was then dehydrated and embedded in paraffin. A 5μm-thick section of the lung was stained with hematoxylin and eosin (H&E) to evaluate lung morphological changes, and masson’s trichrome was used to determine collagen deposition.

### Immunofluorescence staining

Formalin-fixed, paraffin sections (4 μm) of pulmonary tissues fixed with 4% PFA and permeabilized with 0.25% Triton X100 were stained with COL1A1 (Abcam, ab138492) and α-SMA (Abcam, ab7817). Alexa Fluor 488-conjugated anti-mouse IgG (Invitrogen, Carlsbad, CA) and Alexa Fluor 594-conjugated anti-rabbit IgG (Invitrogen, Carlsbad, CA) were used as secondary antibodies. DAPI (Santa Cruz Biotechnology, Heidelberg, Germany) was used to detect nuclei. Leica TCS SP2 confocal laser scanning microscope was used for capturing images.

### Quantitative real-time-PCR

In accordance with the manufacturer’s instructions, total RNA was isolated from lung tissue using an RNA Purification Kit (EZ Bioscience, USA). Prime Script RT Master Mix (Takara, China) was used for complementary DNA synthesis. An iTaq universal SYBR Green Supermix (Bio-Rad, Hercules, CA, USA) was used for real-time PCR with a Light Cycler 480 real-time PCR system (Roche, USA). In each sample, cDNA amounts were normalized with GAPDH. The primer sequences used in this study are listed in **Supplementary Table 1**. The 2−ΔΔCt method was employed to calculate relative expression levels.

### EV isolation, characterization

The supernatant from BALF was collected after centrifuging them at 500 g, 2500 g, and 12,000 g sequentially. Isolated pellets were resuspended in 20uL PBS and stored at 4°C for further use after ultracentrifugation at 100,000 g for 70 minutes.

Samples were placed on a carbon-coated copper mesh for 90 s before being stained with uranyl acetate dye solution for 30 s and visualized under a transmission electron microscope (TEM). The NanoSight NS300(Malvern Panalytica, UK) was used to measure the diameter of EV using Nanoparticle tracking analysis (NTA). WB was used to detect the surface marker proteins of EV.

### Isolation and quantification of RNA from BALF-EV

SeraMir Exosome RNA Purification Column kit (System Biosciences, USA) was used to isolate total RNA from BALF-EV. RNA concentration was measured with the Agilent Bioanalyzer Small RNA Assay using the Bioanalyzer 2100 Expert instrument (Agilent Technologies, USA) for each sample. 1.5% agarose gels were used to monitor RNA degradation, contamination and DNA contamination. NanoDrop 2000 Spectrophotometer (Thermo Fisher Scientific, USA) was used for measuring RNA concentration and purity. The Agilent Bioanalyzer 2100 System (Agilent Technologies, USA) was used to assess RNA integrity.

### Library preparation for high‐throughput whole transcriptome sequence

With the Ribo-Zero rRNA Removal Kit (Epicentre, USA), 1.5 g of RNA was used per sample to remove rRNA. Based on the manufacturer’s recommendations, sequencing libraries were generated using NEBNextR UltraTM Directional RNA Library Prep Kit for Illumina (NEB, USA), and index codes were added to attribute sequences to each sample.

### Clustering sequencing and quality control

According to the manufacturer’s instructions, the index-coded samples were clustered through cBot Cluster Generation System using TruSeq PE Cluster Kitv3-cBot-HS (Illumia, USA). Sequencing and generating reads were performed on an Illumina platform after cluster generation. Fastq reads (Raw data) were processed using in-house Perl scripts to remove adapter data, ploy-N data, and low-quality data.Reads containing adapter, reads containing ploy-N, and low-quality reads were removed from raw data of fastq format *via* in-house Perl scripts; Q20, Q30, GC-content, and sequence duplication levels of the clean data were computed. A high-quality set of clean data was used for all downstream analysis.

### NcRNA analysis

The Clean Reads were aligned with GtRNAdb database, Repbase database, Silva database and Rfam database using Bowtie tool software, respectively. Comparing the reads with miRBase’s predictions of known miRNAs allowed us to detect known-miRNAs and new miRNAs. From the mapping results, read counts for each miRNA were obtained.

CIRI (CircRNA Identifier) tools were used to identify circRNA, which scans SAM files twice and collects enough information to determine its identity and characteristics. According to CIRI tools, the number of junctions reads indicates the expression of circRNA.

StringTie was used to assemble the transcriptome from reads mapped to the reference genome, and GFFcompare was used to annotation the assembled transcripts. A FPKM calculation for lncRNA was performed using StringTie.

DESeq2 R package (1.34.0) was used to analyze differential expression ncRNA between Sham and MV groups. Targetscan was used to predict miRNA target mRNA. MiRanda was used to predict circRNA target mRNA and lncRNA target mRNA.

### NcRNA target genes functional enrichment analysis

Gene function was annotated based on GO (Gene Ontology), KEGG (Kyoto Encyclopedia of Genes and Genomes) and KOG (Eukaryotic Orthologous Groups). In the target gene functional enrichment analysis, Fisher’s exact test was conducted with an adjusted p-value (false discovery rate, FDR) of <0.01 and the absolute value of log2(fold change)> 1 to determine significant changes in differentially expressed ncRNA target genes functions.

### Statistical analysis

We conducted all experiments at least three times and analyzed the data using Graph Pad Prism 9.4 software (USA) to determine the mean and standard deviation (SD). To conclude the means of more than three treatment groups, one-way analysis of variance (ANOVA) was used. In order to compare the differences between the two groups, the student’s t-test (two-tailed) was used. A p-value less than 0.05 (p < 0.05) was considered statistically significant.

## Results

### MV-induced pulmonary fibrosis accompanied by EV release

**Figure 1:**
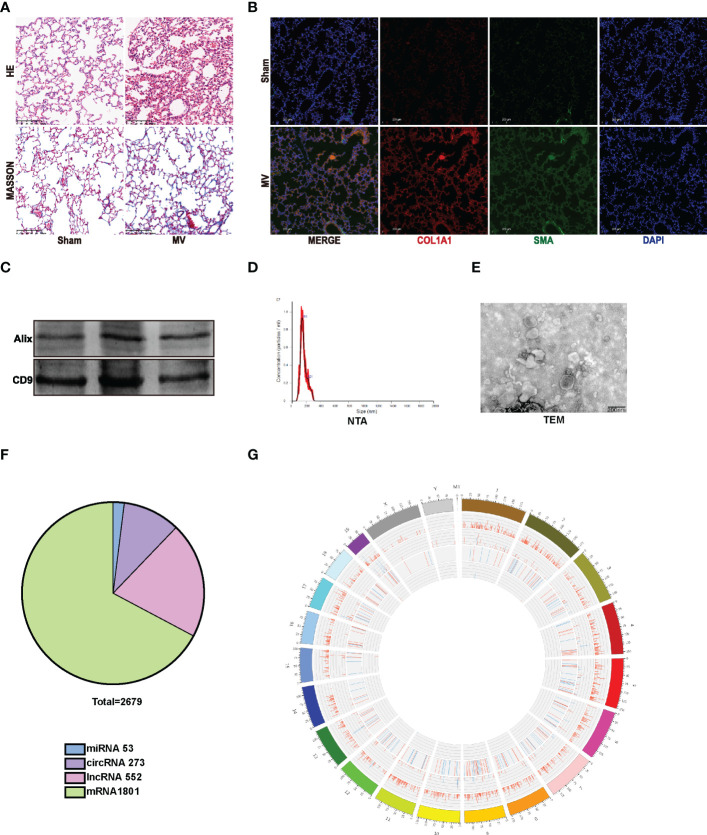
MV-induced pulmonary fibrosis accompanied by EV release. **(A)** lung injury was accessed by Hematoxylin and Eosin staining. Collagen deposition was assessed by Masson’s trichrome staining. Original magnification ×200. Scale bars correspond to 100 μm (n = 6). **(B)** lung tissues were stained with fluorophore-labeled antibodies against COL1A1(Alexa Fluor 594, red), α-SMA (Alexa Fluor 488, green) and nuclei (Alexa Fluor 405, blue) stain by 4’,6-diamidino-2- phenylindole (DAPI). Original magnification ×200. Scale bars correspond to 100 μm (n = 6). **(C–E)**. EV isolated from BALF were determined by WB, NTA and TEM. **(F)** Pie diagram showed the content of the differentially expressed RNAs. **(G)** circus diagram showed the expression level of differentially expressed RNAs according to the location information.

*In vivo*, mice were treated with 20ml/kg MV for 2 hours to establish an MV-induced pulmonary fibrosis model and were observed 7 days later (5). Compared to the Sham group, edema, leukocyte infiltration, and hemorrhage were more obvious in lung tissue of MV mice with H&E staining, and Masson staining showed an increased collagen deposition in MV mice (**Figure 1A**). Immunofluorescence staining confirmed the histologic results: MV significantly increased the percentage of α-SMA^+^ COL1A1 ^+^ cells in mouse lung tissue (**Figure 1B**).

Consequently, we isolated and purified BALF-EVs isolated from Sham and MV group. Western blotting (WB) was used to detect the EV marker protein (**Figure 1C**). Based on nanoparticle tracking analysis (NTA), we confirmed the particles’ strong enrichment in 40–240nm, as well as the multimodal size distribution of EV with a peak diameter of 70-160nm for EV (**Figure 1D**) (11, 12). Furthermore, transmission electron microscopy (TEM) was used to demonstrate purified particles had typical morphological features: membrane-bound, round, and heterogeneous in size (**Figure 1E**).

### Differentially expressed RNAs in BALF EV after MV

High-throughput whole transcriptome sequence was used to determine whether MV affected BALF-EV ncRNA levels. After quality-control and data-filter, a total number of 48850 RNAs, including 26352 mRNAs, 1543 miRNAs,704 circRNAs and 20251 lncRNAs were identified in EVs from BALF of Sham and MV mice. Among the 48850 RNAs, there are 878 differentially expressed ncRNAs including 53 miRNAs, 273 circRNAs, and 552 lncRNAs (**Figure 1F**). According to the location information of RNA, the expression level of different RNA was displayed in circus diagram (**Figure 1G**).

### Identification of miRNA and enrichment analysis of differentially expressed miRNA-targeted mRNAs

**Figure 2:**
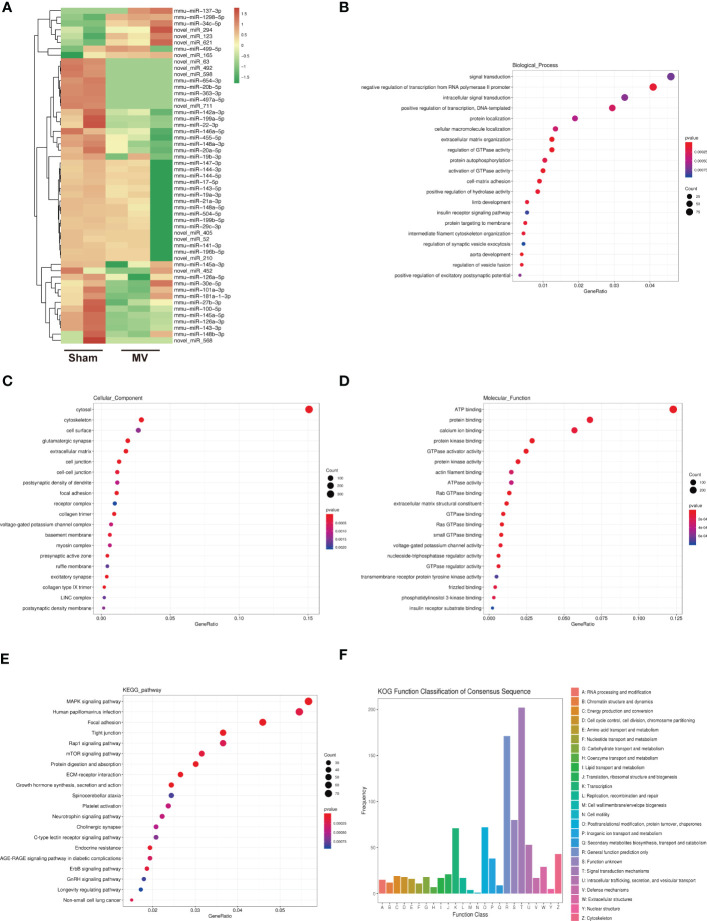
Heatmap of miRNA expression and Enrichment Analysis of differentially expressed miRNA-targeted mRNA **(A)**. heatmap differentially abundant miRNA in the BALF-EV isolated from Sham and MV mice. **(B-D)**. Target genes of differentially expressed miRNA as determined by GO annotation. **(E)**. KEGG pathway analysis of differentially expressed miRNA-targeted mRNA. **(F)**. KOG functional classification of differentially expressed miRNA-targeted mRNA.

In the field of miRNA, particular genes are regulated by miRNA by hybridizing to their mRNAs and promoting their degradation or inhibiting their translation (13). **Figure 2A** showed the result of differentially expressed miRNA in Sham and MV group. In order to investigate the potential functions of differentially expressed miRNAs, their potential target mRNAs were analyzed using TargetScan, and 53 differentially expressed miRNAs were associated with 3105 mRNAs.

To better understand the potential role of these BALF-EV miRNAs after MV, we performed functional annotations and enrichment analysis of these miRNA-targeted mRNAs. GO Functional annotation was divided into biological process (BP), cellular component (CC), and molecular function (MF). GO Functional annotations revealed several enriched functions involved in pulmonary fibrosis (PF): extracellular matrix organization, cell-matrix adhesion, cytoskeleton, collagen trimer, and extracellular matrix constituent (**Figures 2B–D**). The KEGG database provides both functional interpretation and practical applications of genomic data (14). The pathways identified by KEGG were significantly changed (p < 0.05) between Sham and MV group. Among these identified pathways by KEGG, the following have been shown to be associated with PF: MAPK signaling pathway, mTOR signaling pathway and ErbB signaling pathway (**Figure 2E**). KOG function classification was used to classify 53 differentially expressed miRNAs-targeted mRNAs. Among the resultant 26 KOG classifications, the following were involved in PF: Cytoskeleton (43 genes) and Extracellular structures (29 genes) (**Figure 2F**).

### Identification of circRNA and enrichment analysis of differentially expressed circRNA-targeted mRNAs

**Figure 3:**
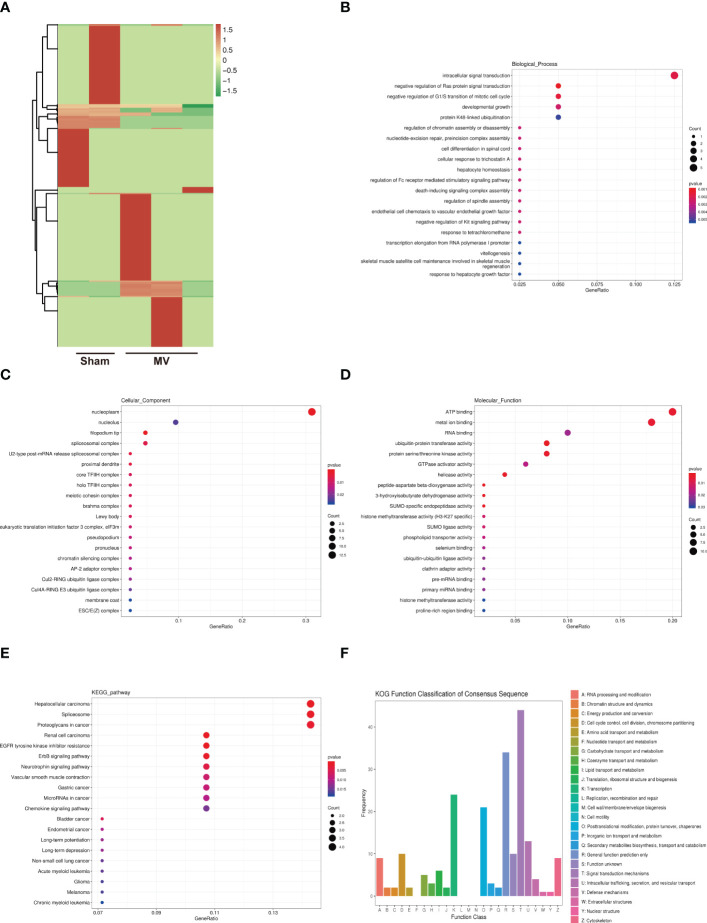
Heatmap of circRNA expression and Enrichment Analysis of differentially expressed circRNA-targeted mRNA **(A)**. heatmap differentially abundant circRNA in the BALF-EVs isolated from Sham and MV mice. **(B-D)**. Target genes of differentially expressed circRNA as determined by GO annotation. **(E)**. KEGG pathway analysis of differentially expressed circRNA-targeted mRNA. **(F)**. KOG functional classification of differentially expressed circRNA-targeted mRNA.

CircRNA is highly conserved in biological evolution and have important biological functions such as miRNA or protein inhibitor, protein function regulator and self-translation (15). **Figure 3A** showed the result of differentially expressed circRNAs in Sham and MV group. To better understand these BALF-EV circRNAs after MV, their potential target mRNAs were analyzed with Miranda. To better understand the potential role of these BALF-EV circRNAs after MV, we performed functional annotations and enrichment analysis of these circRNA-targeted mRNAs.

We found that 273 differentially expressed circRNAs were associated with 241 mRNAs. GO functional annotations revealed several enriched functions involved in PF: developmental growth and meiotic cohesion complex (**Figures 3B–D**). KEGG analysis showed ErbB signaling pathway was involved in PF (**Figure 3E**). KOG function classification was used to functionally classify 273 differentially expressed circRNAs-targeted mRNAs. Among the resultant 25 KOG classifications, the following were found to be involved in PF: Signal transduction mechanisms (44 genes), Cytoskeleton (9 genes), and Extracellular structures (1 gene) (**Figure 3F**).

### Identification of lncRNA and enrichment analysis of differentially expressed lncRNA -targeted mRNAs

**Figure 4:**
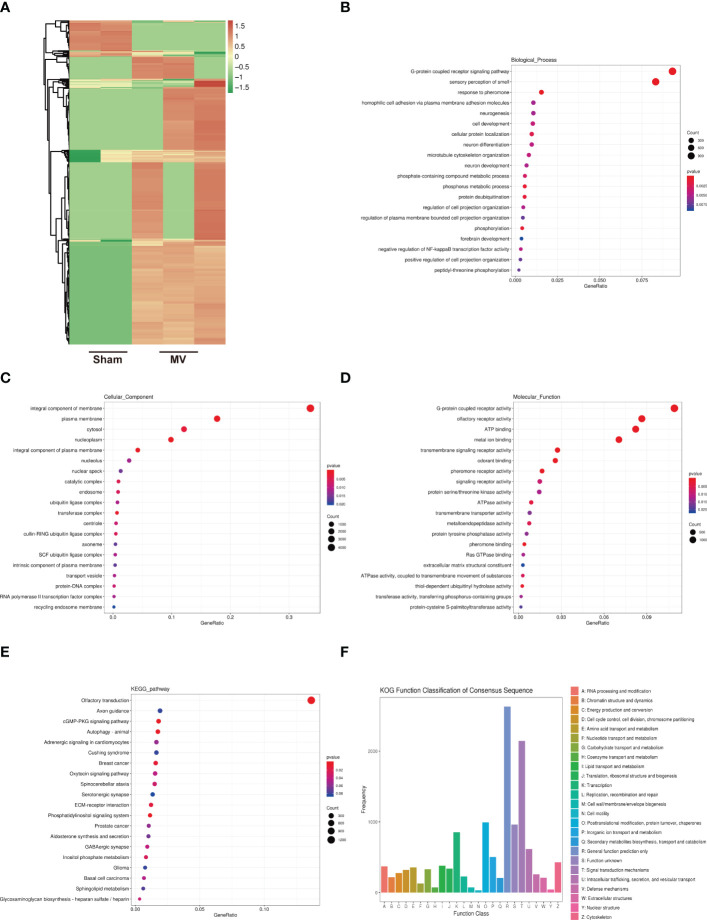
Heatmap of lncRNA expression and Enrichment Analysis of differentially expressed lncRNA-targeted mRNA **(A)**. heatmap differentially abundant lncRNA in the BALF-EVs isolated from Sham and MV mice. **(B-D)**. Target genes of differentially expressed lncRNA as determined by GO annotation. **(E)**. KEGG pathway analysis of differentially expressed lncRNA-targeted mRNA. **(F)**. KOG functional classification of differentially expressed lncRNA-targeted mRNA.

**Figure 4A** showed the result of differentially expressed lncRNAs in Sham and MV group. To better understand these BALF-EV lncRNAs after MV, their potential target mRNAs were analyzed with Miranda.

552 differentially expressed lncRNAs were associated with 20528 mRNAs. GO functional annotations revealed several enriched functions involved in MVPF: homophilic cell adhesion *via* plasma membrane adhesion molecules, cell development, microtubule cytoskeleton organization, regulation of cell projection organization and extracellular matrix structural constituent (**Figures 4B–D**). KEGG pathway analysis identified the following were found to be involved in fibrosis: CGMP-PKG signaling pathway and ECM-receptor interaction (**Figure 4E**). KOG function classification was used to functionally classify 552 differentially expressed lncRNAs-targeted mRNAs. Among the resultant 25 KOG classifications, the following were found to be involved in PF: “Signal transduction mechanisms” (2145 genes), “Intracellular trafficking, secretion, and vesicular transport” (615 genes), “Cytoskeleton” (427 genes) and “Extracellular structures” (210genes) (**Figure 4F**).

### Crosstalk between differentially expressed ncRNA-targeted mRNA

**Figure 5:**
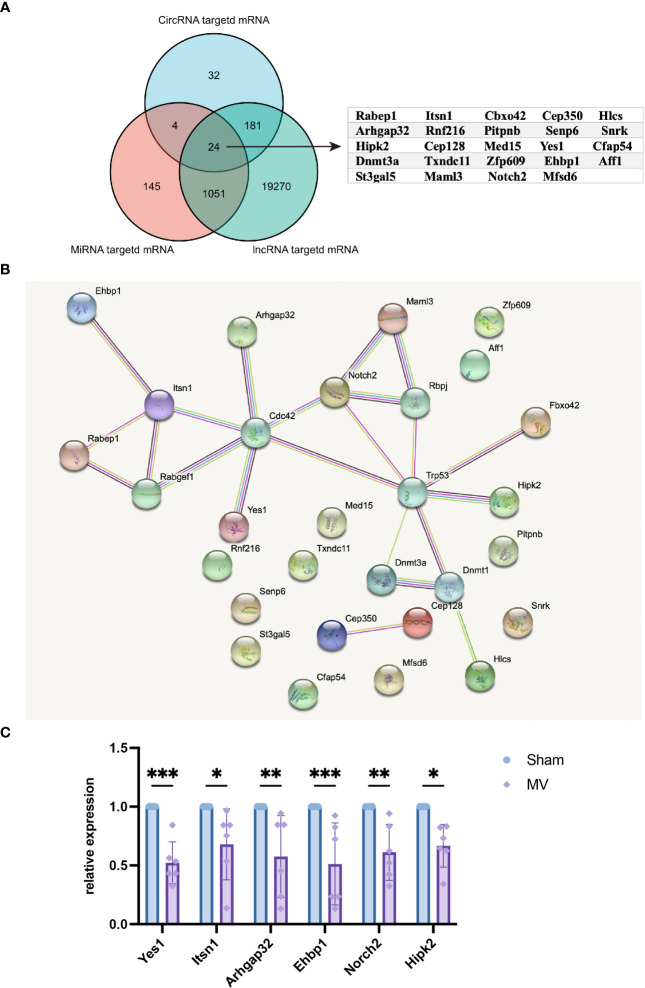
Crosstalk between differentially expressed ncRNA-targeted mRNA **(A)** Venn diagram showed the interaction of ncRNA-targeted mRNA. **(B)** PPI networks of the key genes. **(C)** mRNA expression of key genes in pulmonary tissue was determined by qRT-PCR. *p < 0.05, **p < 0.01, ***p < 0.001.

To better understand the BALF-EV effects, we aimed to find the crosstalk between differentially expressed miRNA-targeted mRNAs, differentially expressed lncRNA-targeted mRNAs and differentially expressed circRNA-targeted mRNAs. Finally, we found 24 common key genes as shown in **Figure 5A**. According to STRING database analysis, PPI networks of the key genes were constructed as shown in **Figure 5B** respectively. As shown in **Figure 5C**, qRT-PCR indicated the level of Yes1, Itsn1, Arhgap32, Ehbp1, Norch2 and Hipk2 were downregulated in mouse lung tissue after MV.

## Discussion

Mechanical ventilation (MV), as an effective treatment for respiratory failure, could also induce VILI and pulmonary fibrosis, which increases mortality in mechanically ventilated ARDS patients (16) and greatly reduces the long-term quality of life of ARDS survivors (17, 18). The pathophysiological mechanism of VILI and MVPF might be the result of interactions between the ventilator and the injured lung which is what we called “barotrauma, volutrauma, atelectrauma and biotrauma” (19). Nevertheless, the specific molecular regulatory mechanism remains unclear. In this study, we applied next-generation sequencing and bioinformatics analysis to identify differentially expressed ncRNAs and related signaling pathways involved in the process of MVPF. We finally identified 53 miRNAs, 273 circRNAs and 552 lncRNAs which showed significant differential expression in mice BALF EVs of two groups. These differentially expressed ncRNAs-targeted mRNAs were closely related with PF by GO, KEGG and KOG analysis, suggesting that ncRNAs alterations in BALF EVs might contribute to the pathogenesis of MVPF. Besides, we also found 6 key genes that may be potential therapeutic targets for MVPF treatment in the future.

In recent decades, researches have shown that cells communicate with each other mainly through EV release, which can act directly on adjacent cells (paracrine signaling) or end up in circulating body fluids and have effects on distant organs (endocrine signaling) (20). EV contains both mRNA (21) and ncRNA including lncRNA (22), miRNA and circRNA (23) that have diverse roles in various biological processes. Many biological processes are regulated by these specialized RNAs, such as RNA processing, RNA silencing as well as protein degradation and trafficking (24). NcRNAs have increasingly been shown to be embedded into EV, protecting from RNase degradation, and absorbed by adjacent or distant cells where they could subsequently modulate host cell function (9). A disruption in cells’ physiological mechanisms may result in a wide variety of diseases like pulmonary fibrosis which is caused by upregulation or downregulation of specific ncRNAs (25).

The present understanding of the function of miRNA in pulmonary fibrosis was outlined by Kusum et al. in 2011. Idiopathic Pulmonary Fibrosis (IPF) was proved to be mediated by let-7, miR-21, miR-29, and miR-155, which shed light on phenotypic regulation of IPF (26). A review in 2016 by Huimin Li revealed that miRNAs were involved in IPF through intricate pathways (27). As for circRNAs, Zhang et al. reported that circHIPK3 could induce IPF by fibroblast-to-myofibroblast transition (FMT) and fibroblast proliferation in male C57BL/6 J mice, WI-38 cells and HEK-293T cells (28). CircRNAs have also been proved to play a role in the process of pulmonary fibrosis by mediating the endothelial-to-mesenchymal transition (EndMT) process (29) (30). What’s more, lncRNAs could also participate in the development of pulmonary fibrosis through EndMT process (29, 31, 32). However, ncRNA changes of MV-treated BALF EVs attract little attention, so we focus on BALF-EV ncRNA alterations, which might be the potential biomarker or therapeutic target for MVPF. We used next-generation sequencing to compare the expression profiles for the whole transcriptome in BALF-EVs isolated from Sham and MV mice. We then found 878 differentially expressed ncRNAs including 53 miRNAs, 273 circRNAs, and 552 lncRNAs. In order to illustrate the potential functions of these differentially expressed miRNAs, circRNAs and lncRNAs, their target mRNAs were analyzed respectively. TargetScan revealed that 53 differentially expressed miRNAs were predicted to target 3105 mRNAs. MiRanda revealed that 552 differentially expressed lncRNAs were predicted to target 20528 mRNAs while 273 differentially expressed circRNAs were associated with 241 mRNAs. Further, we used GO enrichment analysis to functionally annotate ncRNA-targeted mRNAs. Among these terms, the most highly represented and enriched process were “extracellular matrix organization”, “cytoskeleton”, and “cell-matrix adhesion”, which are strongly related to pulmonary fibrosis. KEGG pathway analysis also showed that these ncRNA-targeted mRNAs were enriched in known fibrosis-related signaling pathways. KOG was used to functionally classify ncRNA-targeted mRNAs, and we found that “Cytoskeleton” and “Extracellular structures” were the most important classifications. The GO, KEGG pathway analysis and KOG classification showed that ncRNA-targeted mRNAs were associated with fibrosis related signaling pathways and biological processes, implying that BALF-EV ncRNAs might be crucial in regulating the progress of MVPF.

In this study, we also screened key genes in BALF-EVs through crosstalk between miRNA-targeted mRNAs, lncRNA-targeted mRNAs and circRNA-targeted mRNAs and get 24 common key genes. We then constructed a PPI network and then we validate the expression of these genes in pulmonary tissue by qRT-PCR. Our findings provide a new perspective on the mechanism of MVPF, and it will be essential to apply interventions in these key genes to find the most promising therapeutic targets or biomarkers in the future. Besides, further studies should clarify that how these involved BALF ncRNAs interact and contribute to the development of MVPF.

## Conclusion

Changes in BALF-EV ncRNAs may contribute to MVPF. Identification of key target genes involved in the pathogenesis of MVPF could lead to interventions that slow or reverse fibrosis progression.

## Data availability statement

The datasets presented in this study can be found in online repositories. The names of the repository/repositories and accession number(s) can be found below: PRJNA867324 (SRA).

## Ethics statement

The animal study was reviewed and approved by The Animal Care and Use Committee of Ren Ji Hospital, Shanghai Jiao Tong University School of Medicine.

## Author contributions

RT and YH performed the experiments and wrote the paper. SM, YZ and JF: Figure arrangement and project administration. TJ, BD and SX: Resources. YG: Project administration. QX and ZH: Supervision. All authors approved the final version of the paper. All authors contributed to the article and approved the submitted version.
