# Supplementary material for: Non-coding RNA alterations in extracellular vesicles from bronchoalveolar lavage fluid contribute to mechanical ventilation-induced pulmonary fibrosis
Source: Front Immunol. 2023 Mar 13;14:1141761. doi: 10.3389/fimmu.2023.1141761 (PMC10040560; doi:10.3389/fimmu.2023.1141761)
Supplement: Supplementary file 1 [file Table_1.docx]

**Supplementary Table S1. Primers’ sequences used in qRT-PCR**

| ID | Sequence(forward) | Sequence(Reverse) |
| --- | --- | --- |
| Rabep1 | CTTCGTTCTGTCGTGATGCC | ACTGGTTGTGTTGTTGTCGTT |
| Ehbp1 | CAGCGTGTGGGAAAACATGC | TGTCCAAACAACCACCAGTTTA |
| Itsn1 | CACCTTTCGGTGGTAGCCTG | AAATCCCGCTATCGGCTTCAG |
| Hlcs | GTCTGCGTCTGCGGAGAAC | ACAGTCAGTCAGGACAGATCG |
| Arhgap32 | ATGAAGTCTCGCCCAACAAAA | CAAACCCAGAATTGAGGAGGTG |
| Yes1 | CACACCGGAAAATCTTACAGAGC | TCCAAAAGGAGTCACCCCTGA |
| Notch2 | GAGAAAAACCGCTGTCAGAATGG | GGTGGAGTATTGGCAGTCCTC |
| Maml3 | GCCGCGAATGGTAGTAGTATC | GAGTGCTATTGGGAGTATTCACG |
| Hipk2 | ATGTGCAAGTTTTCTCCCCTC | CTCGTAAGGTAGGCTTGGGTT |
| Dnmt3a | GATGAGCCTGAGTATGAGGATGG | CAAGACACAATTCGGCCTGG |
| Aff1 | GAAGGAAAGACGCAACCAAGA | GCTCATCGCCTTTTGCAGTC |
| Med15 | CTGGTCAGCCAAATTGAGGAT | TCAGGAACACATGACTCTCCATA |
| Cbxo42 | TGCCGGAAGAGGTTTTGGAG | GCCACACCTTTGATAAGTCGATA |
| Cep350 | ATGTAACCACATCATGGGATGC | CGGGTAGCACTTGCAGACTTC |
| Cep128 | ACCACAGTCGCTTCCTGTCTA | GGTATCTCCCTCGATTGATGCT |
| Zfp609 | AGTGGATGCAAACCCGGTTG | GCACTGGGGTGACAAACTTG |
| Pitpnb | TCCATTCAGTCAAGACCAAGAGA | AACCCTTCTTACGCATTGTTTCT |
| Rnf216 | GCCCATCCTCTAGGAGAGCTT | CCGTTTCTTTCACTAACAGTGGA |
| Senp6 | GTTGTTTGTTTCCCTGGTTTGG | GCACTCGAATCAGTCACAGCTA |
| Snrk | GGGTTCAAGCGAGGATATGATG | AAGTTTGACCACTGCAAAATGAC |
| Cfap54 | CAGACTCTGAGACATCGGTGT | CCTCGGAGTGGGTACATTGC |
| Txndc11 | CTTTTGCCCGTCTCTCAATCA | AGGGTATTTCACACTTAGGTCCT |
| St3gal5 | ATGCCAAGTGAGTTCACCTCT | CTGGGTTCGGGTGTACCATT |
| Mfsd6 | AAATATGTGCTTGCTGATCCCTT | AAAGAGAGCCATAGGCAGAGTA |
| GAPDH | AGGTCGGTGTGAACGGATTTG | GGGGTCGTTGATGGCAACA |
